# RAGE Genetic Polymorphisms Are Associated with Risk, Chemotherapy Response and Prognosis in Patients with Advanced NSCLC

**DOI:** 10.1371/journal.pone.0043734

**Published:** 2012-10-05

**Authors:** Xiang Wang, Enhai Cui, Huazong Zeng, Feng Hua, Bin Wang, Wei Mao, Xueren Feng

**Affiliations:** 1 Huzhou Central Hospital, Huzhou, Zhejiang, China; 2 School of Life Sciences and Technology, Tongji University, Shanghai, China; University of Porto, Portugal

## Abstract

**Aim:**

To explore the association between genetic polymorphisms of the receptor for advanced glycation end-products (RAGE) and susceptibility, chemotherapy response rate and prognosis of non-small cell lung cancer (NSCLC).

**Method:**

This is a prospective study in which 562 patients with NSCLC and 764 healthy controls were enrolled. Three RAGE genetic polymorphisms, namely, −429T/C, −374T/A and 82G/S were genotyped. Platinum-based chemotherapy was given to 432 subjects with advanced inoperable NSCLC and their responses to chemotherapy were evaluated.

**Results:**

All the polymorphic genotypes of RAGE polymorphisms were associated with susceptibility for NSCLC. Only the 82G/S polymorphisms denoted a significant difference between responders and non-responders to chemotherapy. The 82SS genotype and 82S allele distribution not only increased the NSCLC risk, but also was associated with a lower chemotherapy response rate and poor prognosis, indicated by overall survival and progression free survival.

**Conclusion:**

The 82G/S genetic polymorphism of RAGE gene might be used as a genetic marker to screen for patients sensitive to thermotherapy and to predict the prognosis of NSCLC.

## Introduction

Lung cancer is currently the most widespread cancer worldwide. Non-small cell lung cancer (NSCLC) accounts for 80% of all lung cancers. NSCLC has been viewed as a disease caused by smoking and various forms of environmental/occupational exposure, however, many studies suggest that genetic factors also contribute to the incidence of NSCLC [1]. Despite the progress in treatment, the prognosis of NSCLC remains very poor, especially for those with advanced NSCLC. Platinum-based chemotherapy is the first line chemotherapy for those with advanced inoperable NSCLC; however, the chemotherapy response rate is quite different among patients [2], [3], [4]. Clinically, about one-third of NSCLC patients achieves remission from standard chemotherapy, another one-third maintains stable disease and the remaining one-third develops progressive disease [5]. The detailed mechanism under which the NSCLC patient responds differently to chemotherapy remains unclear. Recent studies showed that genetic factors may play an important role in determining the chemotherapy response in cancer patients receiving chemotherapy [6], [7], [8].

The receptor for advanced glycation end-products (RAGE) is a transmembrane receptor of the immunoglobulin superfamily, which contributes to growth, survival, and metastatic spread of cancers [9], [10]. RAGE is expressed at a low basal level in the majority of healthy adult tissues, however, its expression is remarkably high in lung tissue, suggesting that RAGE may have certain functions in the lung distinct from the other tissues [11]. Down-regulation of RAGE may be considered as a critical step in tissue reorganization and the formation of lung tumors[12]. A recent study proposed that serum soluble RAGE (sRAGE) may act as an effective and convenient diagnostic biomarker for lung cancer [13].

To date, several genetic variants have been identified in the RAGE gene, including −429 T/C, −374T/A and 82G/S, which may affect the expression or function of RAGE [14], [15], [16]. Pervious studies reported positive associations between the variants of RAGE gene and various types of cancers, including gastric cancer [17], pancreas cancer [18] and breast cancer [19]. However, surprisingly, no study regarding the role of RAGE genetic variants in NSCLC was reported. In the present study, we performed a case-control study in a Chinese cohort to test the possible genetic association between RAGE genetic variants and the susceptibility to NSCLC; meanwhile, we also tested the role of RAGE polymorphisms in determining the chemotherapy responses in advanced NSCLC subjects.

## Methods

### Enrollment

We recruited 562 patients who were histologically diagnosed as advanced NSCLC in our hospital from September 2003 to January 2008. We also enrolled 764 sex and age-matched healthy volunteers as controls. Clinical characteristics and data, including sex, age, smoking status and family history of cancer were obtained. The cancer stage was determined based on the International Union Against Cancer (UICC) TNM classification. [20] This study was approved by the institutional review board of our hospital and written consent was obtained from all subjects.

To avoid the potential influence of poor clinical conditions on chemotherapy response, the following eligibility criteria had to be met: normal blood chemistries (hemoglobin >9 g/dl, neutrophil count >1500/mm3 and platelet count >100 000/mm3), hepatic function (bilirubin <1.5 times the normal upper limit, aspartate aminotransferase and alanine aminotransferase <2 times the normal upper limit) and renal function (Creatine clearance rate >50 ml/s), and normal electrocardiogram at the beginning of treatment. Exclusion criteria included symptomatic brain metastases, spinal cord compression, uncontrolled massive pleural effusion and previously received chemotherapy and other chronic diseases.

### Chemotherapy regimens and therapeutic effect evaluation

Of enrolled 562 NSCLC patients, 407 were advanced inoperable NSCLC (stage III and IV). They received platinum-based chemotherapy as first-line chemotherapy as shown in table 1. Concrete dosage: cisplatin (DDP) 75 mg/m2 on day 1; Carboplatin AUC (area under the concentration–time curve) 6.0 mg/ml/min on day 1; docetaxel (DOC) 75 mg/m2 on day 1 (kept for 1 h); gemcitabine (GEM) 1,250 mg/m2 on day 1 and day 8; vinorelbine (NVB) 25 mg/m2 on day 1 and day 8; pemetrexed (PEM) 500 mg/m2 on day 1. All chemotherapeutic drugs were given intravenously, and the treatment cycles were repeated every 3–4 weeks. Patient responses to treatment were determined after four cycles by the WHO criteria, which classify the response into four categories: complete response (CR), partial response (PR), stable disease (SD), and progressive disease (PD). CR was defined as complete disappearance of all measurable lesions. PR required at least 50% reduction in measurable lesions. Patients with SD had less than a 50% decrease or no more than a 25% increase in the size of measurable lesions. PD was assigned to patients when measurable lesions increased by more than 25% or new lesions appeared. [21], [22], [23]

**Table 1:** Summarized the clinical characteristics of NSCLC cases and controls.

Characteristics Patient	Case(562)		Control (764)		P
**Age (years)**					
<60	342	60.85%	465	60.86%	NS
>65	220	39.15%	299	39.14%	
**Gender**					
Male	326	58.01%	440	57.59%	NS
Female	236	41.99%	324	42.41%	
**Smoke status**					
non-smokers	306	54.45%	474	62.04%	NS
smoker	256	45.55%	290	37.96%	
**Histology**					
Squamous cell carcinoma	302	53.74%			
Adenocarcinoma	260	46.26%			
**Stage**					
I	40	7.12%			
II	115	20.46%			
III	266	47.33%			
IV	141	25.09%			
**Differentiation**					
Well	139	24.73%			
Moderate	265	47.15%			
Poor	158	28.11%			
**Chemotherapy regimens** (n = 407)					
DDP/CBP+DOC	156	38.33%			
DDP/CBP+GEM	164	40.29%			
DDP/CBP+NVB	76	18.67%			
DDP/CBP+PEM	11	2.70%			

For data analysis, CR and PR were combined as responders, and SD and PD were grouped as non-responders. After the treatment, the patients were followed up. Overall survival (OS) and progression free survival (PFS) were the end points in this study. OS was calculated from the date of chemotherapy to the date of last follow-up or death from any cause. PFS was defined as the interval between the date of chemotherapy and the date of confirmed relapse. Patients who were not deceased were censored at the last date they were known to be alive based on date of last contact.

### Sample Collection and Genotyping

Venous blood (10 ml) was collected from each patient into tubes containing 50 mmol of EDTA per liter, and genomic DNA was isolated with DNA blood Mini kit, according to manufacturer's instructions. The gene encoding RAGE is located on chromosome 6p21.3 and comprises 11 exons (spanning 3.27 kb). Four polymorphisms of the RAGE gene, −429 T/C, −374 T/A and 82G/S, were determined from DNA extracted from a sample of peripheral blood. For amplification of the region containing the −374 T/A and −429 T/C polymorphisms, the following primers were used: forward primer 5′ GGG GCA GTT CTC TCC TCA CT 3′ and reverse primer 5′ GGT TCA GGC CAG ACT GTT GT 3′. Polymerase chain reaction (PCR) amplification was conducted in a 25-µL volume containing 100 ng of genomic DNA and 12.5 pmol of each primer. Annealing temperature was 59.5°C and final extension occurred at 72°C for 7 min. Restriction analysis was performed with all PCR product using 3 units of restriction nucleases, AluI for Gly82Ser and MfeI for −374 T/A polymorphisms overnight at 37°C. The restriction products were directly separated by electrophoresis in 3 percent agarose gel, and visualized in ultraviolet (UV) light after ethidium bromide staining. Digestion with MfeI revealed fragments of 215 and 35 bp for the wild-type allele −374 T and 250 bp for the mutated allele −374 A. After the digestion reaction with AluI, fragments of 250 bp for the −429 T allele (wild type) and 88 and 162 bp for the −429 C allele were detected. The Gly82Ser polymorphism in exon 3 of the RAGE gene was amplified by PCR using primers 5′ GTA AGC GGG GCT CCT GTT GCA 3′ and 5′ GGC CAA GGC TGG GGT TGA AGG 3′. The 397-bp PCR product was digested by AluI enzyme. Genotypes for the Gly82 and Ser82 alleles were detected as bands corresponding to fragment sizes of 248 and 149 bp for the wild-type allele Gly82, and 181, 67, and 149 bp for the minor allele Ser82. About 10% of the samples were randomly selected to do the repeated assays, and the results were 100% concordant. Two researchers, blinded to the clinical data, scored the genotypes independently.

### Statistical Analyses

χ2 or fisher tests were used to compare genotype frequency and demographic distributions between cases and controls. Multiple logistic regression analyses was used to evaluate if each SNP was independently associated with NSCLC when adjusted for the potential confounding effects of important clinical variables. The odds ratios (OR) and 95% confidence intervals (CIs) were calculated. The associations between the RAGE haplotypes and NSCLC risk were analyzed. The D′ value and r2 for the studied 3 SNPs were calculated with the SHEsis software [24]. The differences in OS and PFS across different genotypes were compared using the log-rank test with adjustment for age, sex, smoking status, cancer stage, differentiation status and chemotherapy regimens. A Cox regression model was performed to obtain the adjusted hazard ratio (HR) and 95% CI for potential prognostic factors for OS in NSCLC patients. P<0.05 was considered statistically significant. All analyses were performed by using SPSS software (Statistical Package for the Social Sciences, version 16.0, SPSS Inc, Chicago, IL, USA).

## Results

### Participants' characteristics

Table 1 shows demographic and clinical characteristics of all subjects in the study. There were no significant differences in sex and age distribution. However, there was a higher proportion of smokers among patients with NSCLC than among controls (P = 0.006). 40 patients (7.12%) were at tumor stage I, 115 patients (20.46%) at stage II, 266 patients (47.33%) at stage III (A and B) and 141 (25.09%)were at stage IV. 302 (53.74%) patients had squamous cell carcinoma, 260 (46.26%) had adenocarcinoma. 139 patients (24.73%) had well differentiated tumors; 265 patients (47.15%) had moderately differentiated tumors and 158 (28.11%) had poorly differentiated tumors. 407 patients with inoperable stage III and IV received platinum-based chemotherapy:156 received DDP/CBP+DOC (38.33%);164 received DDP/CBP+GEM (40.29%);76 received DDP/CBP+NVB (18.67%) and 11 received DDP/CBP+PEM (2.70%).

### RAGE polymorphism between NSCLC cases and controls

The genotype and allele frequencies of RAGE polymorphisms between NSCLC cases and controls are shown in Table 2. The genotype frequencies of the all polymorphisms in controls were in Hardy-Weinberg equilibrium (all P>0.05). There were significant differences in the genotype distributions and allele frequencies of all the three RAGE polymorphism between NSCLC cases and controls (All P<0.05, table 2). By multivariate analyses, markedly higher risk for NSCLC was observed in 82GS and 82SS carriers of 82G/S (OR = 2.08 and 1.72, both P<0.005, with GG as reference) after adjustment with age, sex, smoking status, histology, differentiation and stage. In contrast, the TT genotype of −374T/A and CC of −429 T/C represented significantly reduced risk for NSCLC after adjustment with the above mentioned variables (OR = 0.63 and 0.67, both P<0.05, with −374 AA and −429TT as reference, respectively).

**Table 2:** The genotype and allele frequencies of RAGE polymorphisms between NSCLC cases and controls.

		Case		Control		OR	95%CI		X^2^	P
		N	%	N	%					
−374T/A	AA	139	24.73%	177	23.17%	1				
	AT	330	58.72%	399	52.23%	1.05	0.81	1.37	0.15	0.007
	TT	93	16.55%	188	24.61%	0.63	0.45	0.88	7.43	0.006
	A	608	54.09%	753	49.28%	1				
	T	516	45.91%	775	50.72%	0.82	0.71	0.96	6	0.0014
82G/S	GG	84	14.95%	197	25.79%	1				
	GS	360	64.06%	406	53.14%	2.08	1.55	2.79	24.63	<0.001
	SS	118	21.00%	161	21.07%	1.72	1.21	2.44	9.34	0.002
	G	528	46.98%	800	52.36%	1				
	S	596	53.02%	728	47.64%	1.44	1.06	1.45	7.5	0.006
−429T/C	TT	201	35.77%	229	29.97%	1				
	TC	274	48.75%	387	50.65%	0.81	0.63	1.03	2.97	0.085
	CC	87	15.48%	148	19.37%	0.67	0.48	0.93	5.85	0.016
	T	676	60.14%	845	55.30%	1				
	C	448	39.86%	683	44.70%	0.82	0.71	0.96	6.21	0.013

### RAGE haplotypes and NSCLC risk

The associations between the RAGE haplotypes and NSCLC risk were analyzed in this study. The D′ value and r ^2^ for the studied polymorphisms were calculated with the SHEsis software [24]. All the polymorphisms at three loci were in strong LD (all D′>0.8). The estimated haplotype frequencies of the RAGE polymorphisms are shown in Table 3. The haplotypes A_−374_G_82_C_−429_ and T_−374_G_82_T_−429_ represented protective effects for developing NSCLC (all OR<1 and all P<0.05). Meanwhile, the A_−374_S_82_T_−429_ haplotype showed a higher risk for developing NSCLC (OR = 1.810, P<0.001).

**Table 3:** The estimated haplotype frequencies of the RAGE polymorphisms between NSCLC and controls.

−374T/A	82G/S	−429T/C	Case(freq)	Control(freq)	Chi2	P	Odds Ratio [95%CI]
A	G	C	115.69(0.103)	203.33(0.133)	5.56	0.018	0.747 [0.587∼0.953]
A	G	T	173.02(0.154)	244.56(0.160)	0.183	0.669	0.955 [0.772∼1.180]
A	S	C	140.31(0.125)	171.95(0.113)	0.942	0.332	1.125 [0.887∼1.427]
T	S	C	139.37(0.124)	180.42(0.118)	0.214	0.643	1.057 [0.835∼1.338]
T	G	T	99.92(0.089)	171.70(0.112)	3.879	0.049	0.771 [0.595∼0.999]
A	S	T	262.76(0.234)	256.79(0.168)	17.753	<0.001	1.810 [1.246∼2.831]

### RAGE SNPs and chemotherapy response status

Of the 407 patients who received platinum-based chemotherapy for advanced inoperable stage III and IV NSCLC, 133 (32.7%) were chemotherapy responders (CR+PR) and 274 (67.3%) were non-responders (SD+PD). There were no significant difference in mean age, gender distribution, smoking status, histology status and chemotherapy agent between responders and non-responders (data not shown). Of all the studied RAGE polymorphisms, only 82G/S were significantly different between responders and non-responders. Responder had a markedly lower SS and GS genotype than non-responders (table 4). The S allele carriage was lower in responders than non-responders (37.97% vs. 48.36%). after the adjustment with age, sex, smoke status, histology, differentiation and stage, multivariate regression analyses showed the 82GS and 82SS carriers markedly lower rate of thermotherapy response in than 82GG carriers (OR = 0.59 and OR = 0.45, both P<0.005). The S allele carriage represented a lower rate of chemotherapy response as well (OR = 0.65, P = 0.005). There was no significant association between the other two genotypes of RAGE polymorphisms and chemotherapy response was found (data not shown).

**Table 4:** The genotype and allele frequencies of RAGE polymorphisms between chemotherapy responders and non-responders.

		Responder		Non-responder		global	adjusted	95%CI		X2	Adjusted P
						P	OR				
−374T/A		N	%	N	%						
	TT	18	13.53%	45	16.42%	0.167	1				
	TA	82	61.65%	182	66.42%		1.13	0.61	2.06	0.15	0.7
	AA	33	24.81%	47	17.15%		1.76	0.87	3.55	2.47	0.116
	T	118	44.36%	272	49.64%		1				
	A	148	55.64%	276	50.36%		1.24	0.92	1.66	2	0.158
−429T/C	TT	27	20.30%	42	15.33%	0.338	1				
	TC	82	61.65%	188	68.61%		0.68	0.39	1.17	1.93	0.164
	CC	24	18.05%	44	16.06%		0.85	0.42	1.7	0.22	0.642
	T	136	51.13%	272	49.64%		1				
	C	130	48.87%	276	50.36%		0.94	0.7	1.26	0.16	0.69
82G/S	GG	53	39.85%	73	26.64%	0.019	1				
	GS	59	44.36%	137	50.00%		0.59	0.37	0.95	4.84	0.028
	SS	21	15.79%	64	23.36%		0.45	0.25	0.83	6.72	0.01
	G	165	62.03%	283	51.64%		1				
	S	101	37.97%	265	48.36%		0.65	0.48	0.88	7.81	0.005

The associations between the clinical variables and PFS as well as OS were studied by log-rank test. Only smoking status slightly affected the median PFS (P = 0.054, Table 5). For OS, our results showed that smoking status, tumor stage and differentiation status significantly influenced the OS during the follow-up period (all P<0.01). The association between the RAGE polymorphisms and PFS and OS were analyzed. We combined the GS and SS carriers in one group, whit the GG carriers as reference. Our data showed that patients with the 82GS+82SS genotype had shorter OS and PFS than the GG carriers (P = 0.018 and <0.001, respectively). Multivariate Cox proportional hazards regression models were performed to estimate the hazard ratios (HR) for overall survival and their 95% CIs, with adjustment for age, sex, smoking status, histology, stage, chemotherapy status. The HR for 82GS+82SS genotype was 1.73 for PFS (95% CI: 1.13–2.58, P = 0.003) and 2.34 for OS (95% CI: 1.93–3.94, P<0.001). The gene polymorphisms of −374T/A and −429T/C did not affect the PFS and OS (data not shown). Besides 82G/S polymorphisms, the other risk factors influencing prognosis included smoking status, chemotherapy response status and cancer stage (table 6).

**Table 5:** The associations between the clinical variables and PFS as well as OS were studied by log-rank test.

Variables	Median PFS	95%CI	P	Median OS	95%CI	P
**Smoke status**						
Non-smokers	5.87	5.46–6.11	0.054	8.94	8.25–10.41	0.004
Smoker	5.6	5.37–5.95		8.18	7.01–9.56	
**Stage**						
III	5.68	5.27–5.67	0.185	8.45	8.23–8.86	0.048
IV	5.65	5.11–5.79		8.37	8.19–8.79	
**Differentiation**						
Well	5.53	5.25–5.78	0.071	9.39	8.51–9.98	<0.001
Moderate+Poor	5.51	5.21–5.82		8.54	8.23–9.16	
82G/S						
GG	5.93	5.22–6.25	0.018	9.49	8.85–10.37	<0.001
GS+SS	5.24	4.88–5.57		8.44	8.13–8.96	

**Table 6:** Adjusted hazard ratio for PFS and OS in NSCLC patients.

			PFS			OS	
		HR	95%CI	P	HR	95%CI	P
**82G/S polymorphism**	82GG	1			1		
	82GS+82SS	1.73	1.13–2.58	0.003	2.34	1.93–3.94	<0.001
**Chemotherapy responder**	Non-Responder	1					
	Responder	5.23	2.76–7.98	<0.001	4.32	2.56–8.46	<0.001
**Smoke**	Non-smoker	1					
	Smoker	1.67	1.32–3.01	0.036	2.45	2.15–4.17	0.002
**Cancer stage**	III	1					
	IV	1.98	1.15–3.05	0.027	2.18	1.78–3.31	0.003

## Discussion

In this study, we investigated the role of genetic polymorphisms of the RAGE gene in determining the susceptibility and chemotherapy response status in subjects with NSCLC. We have two main findings in this study. First, we found that all 3 genetic polymorphism of RAGE gene affect the risk for NSCLC. Secondly, only 82G/S were closely associated with the chemotherapy response and clinical outcome in advanced NSCLC. The 82SS Carriers had significantly lower response rate to chemotherapy and poorer prognosis, indicated by the median PFS and OS periods. To the best of our knowledge, this is the first study documenting the role of RAGE genetic polymorphisms in the risk and chemotherapy response in NSCLC. Our finding may be clinically useful as the 82G/S genetic polymorphisms could be used as a genetic marker for individualizing chemotherapy in advanced NSCLC.

Previous study showed that genetic variants in the RAGE gene could alter expression and function of RAGE, thus affect disease development [25]. Gly82Ser (or G82S) is one of the most frequently studied RAGE gene variants, which leads to a change from glycine to serine within the putative ligand-binding domain of the protein and it has been proposed as a functional polymorphism and associated with enhanced RAGE signaling [26]. Previous studies revealed that RAGE is one of the key factors accelerating tumor progression and metastasis in various types of cancers [27], [28]. A recent study showed that polymorphism of 82G/S was associated with the increased risk of gastric cancer and adjacent organ invasion in younger Chinese subjects [17]. However, in patients with breast cancer, the genotype and haplotypes of RAGE gene polymorphisms were not associated with cancer risk.[19] In the present study, we found that 82G/S variants were significantly associated with the risk of NSCLC in a Chinese cohort after adjustment with age, sex, smoking status, histology, differentiation and stage. Besides 82G/S, the −374T/A and −429 T/C polymorphisms also affect susceptibility to NSCLC. We performed the haplotype analyses and our results showed that the haplotypes A_−374_G_82_C_−429_ and T_−374_G_82_T_−429_ were protective against NSCLC and the A_−374_S_82_T_−429_ increased the NSCLC risk. These findings suggest that the RAGE genetic polymorphisms and haplotypes are associated with NSCLC risk and may be used as a genetic marker for to screen for subjects at risk for NSCLC.

Identification of predictive markers for response to chemotherapy is most clinically warranted to further improve the efficacy of chemotherapy in advanced NSCLC. Several gene polymorphisms have been reported to correlate with the chemotherapy response.[29], [30], [31], [32] To date, only limited candidate genes were discovered in Chinese population. In the present study, we found that the 82G/S polymorphisms significantly affect the chemotherapy response rate in advanced inoperable NSCLC patients. Logistic regression showed that the 82SS carriers had much less chance to response to chemotherapy, suggesting these patients would have poorer clinical outcomes. To testify this point, we had follow-up study which showed that the 82SS carriers had a shorter PFS and OS period than GG carriers, suggesting a prognostic role of 82G/S in patients receiving chemotherapy.

Several limitations in this study need to be addressed. This study was a single-center cohort investigation, and thus, replication studies with large independent cohorts are warranted. Secondly, we did not detect the plasmatic sRAGE or RAGE protein expression in excised cancer tissue of NSCLC subjects, which will provide the direct evidence to support the association between genetic variants of RAGE gene and clinical conditions.
